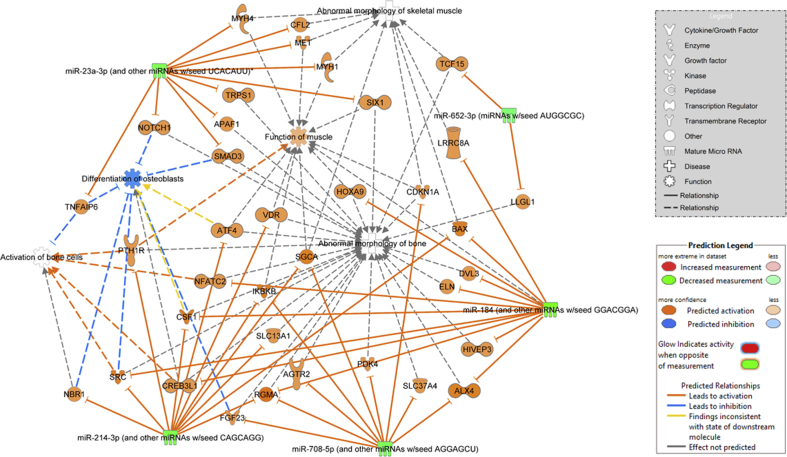# Mouse microRNA signatures in joint ageing and post-traumatic osteoarthritis

**DOI:** 10.1016/j.ocarto.2021.100186

**Published:** 2021-12

**Authors:** Catarina I.G.D. Castanheira, James R. Anderson, Yongxiang Fang, Peter I. Milner, Katarzyna Goljanek-Whysall, Louise House, Peter D. Clegg, Mandy J. Peffers

**Affiliations:** a Musculoskeletal and Ageing Science, Institute of Life Course and Medical Sciences, University of Liverpool, Liverpool, L7 8TX, UK; b Centre for Genomic Research, Institute of Systems, Molecular and Integrative Biology, Biosciences Building, Crown Street, University of Liverpool, Liverpool, L69 7ZB, UK

**Keywords:** Osteoarthritis, Joint ageing, microRNA, Biomarkers

## Abstract

**Objective:**

This study investigated mice serum and joint microRNA expression profiles in ageing and osteoarthritis to elucidate the role of microRNAs in the development and progression of disease, and provide biomarkers for ageing and osteoarthritis.

**Design:**

Whole joints and serum samples were collected from C57BL6/J male mice and subjected to small RNA sequencing. Groups used included; surgically-induced post-traumatic osteoarthritis, (DMM; 24 months-old); sham surgery (24 months-old); old mice (18 months-old); and young mice (8 months-old). Differentially expressed microRNAs between the four groups were identified and validated using real-time quantitative PCR. MicroRNA differential expression data was used for target prediction and pathway analysis.

**Results:**

In joint tissues, miR-140–5p, miR-205–5p, miR-682, miR-208b-3p, miR-499–5p, miR-455–3p and miR-6238 were differentially expressed between young and old groups; miR-146a-5p, miR-3474, miR-615–3p and miR-151–5p were differentially expressed between DMM and Sham groups; and miR-652–3p, miR-23b-3p, miR-708–5p, miR-5099, miR-23a-3p, miR-214–3p, miR-6238 and miR-148–3p between the old and DMM groups. The number of differentially expressed microRNAs in serum was higher, some in common with joint tissues including miR-140–5p and miR-455–3p between young and old groups; and miR-23b-3p, miR-5099 and miR-6238 between old and DMM groups.

We confirmed miR-140–5p, miR-499–5p and miR-455–3p expression to be decreased in old mouse joints compared to young, suggesting their potential use as biomarkers of joint ageing in mice.

**Conclusions:**

MiR-140–5p, miR-499–5p and miR-455–3p could be used as joint ageing biomarkers in mice. Further research into these specific molecules in human tissues is now warranted to check their potential suitability as human biomarkers of ageing.

## 1 Introduction

Osteoarthritis (OA) is a progressive joint disease and an enormous burden for the health-care system and society [1]. OA is mainly characterised by destruction and loss of articular cartilage; notwithstanding, it is a whole joint disease that often includes variable degrees of synovial inflammation, alterations and damage to the joint capsule and soft tissues and hypertrophic changes of bone and subchondral bone [2]. The development of effective therapeutics for OA has been limited by inadequate early diagnosis; OA is characterised by a non-symptomatic phase that if promptly identified would enable early intervention and potentially prevent the disease.

The onset of OA in any given individual is usually the result of a complex interplay of systemic and local risk factors such as age, sex, genetics, obesity, diet, joint anatomy and physical activity, among others. Of all risk factors, age is one of the strongest predictors of OA [3]. Ageing is characterised by a gradual deterioration of all tissue cellular functions, with a global reduction in resistance to stress and concomitant increase of systemic levels of pro-inflammatory mediators [4]. Ageing promotes alterations in the homeostatic balance of joints, affecting their functional capacity. Age-related joint changes such as chondrocyte senescence prompt imbalances in catabolic and anabolic signalling, leading to overproduction of matrix degrading enzymes predisposing to OA development [5]. Previous mouse model studies have demonstrated that age affects the response to surgically induced OA, with older mice having increased cartilage degradation severity, greater thickening of the subchondral bone plate and increasing size of osteophytes post-surgery [6], along with altered patterns of gene expression [7]. Therefore, understanding how joints change during OA progression as a function of age may allow for the identification of markers of joint ageing, providing risk indicators for the onset of OA.

Substantial research has been undertaken in epigenetic regulation in ageing and OA, and interest for the field of microRNAs is growing [8]. These small non-coding RNA molecules affect numerous cellular processes and cell signalling pathways through post-transcriptional regulation of gene expression and RNA silencing [9]. As with several other diseases, OA is accompanied by altered expression of microRNAs. These molecules are likely to contribute to disease progression and may act as circulating biomarkers for disease, which is particularly attractive for early OA detection [9].

Although age-related changes in cartilage have been identified as critical factors in OA development, few studies interrogating microRNA expression in ageing joint tissues are reported. Our group has previously described differential transcriptional signatures associated with ageing in equine cartilage, including increased expression of miR-21 [10]. More recently, we described the effect of ageing on the expression of small non-coding RNAs in equine chondrocytes using small RNA sequencing. MiR-122 and miR-148a were upregulated in old chondrocytes, while miR-143, miR-145 and miR-181b were downregulated [11]. We also demonstrated changes in microRNA expression in aged human knee cartilage, and found miR-126–3p and miR-424–3p to be downregulated in old samples [12].

Here we investigate the microRNA expression profile in ageing and OA mice to further understand the functional significance of microRNAs in the development and progression of disease, as well as provide biomarkers for ageing and OA. We have previously determined the expression patterns of small nucleolar RNAs (snoRNAs) in joint ageing and OA [13]. On par with this, herein we compare microRNA expression of pooled joint tissues (including cartilage, meniscus, subchondral bone and joint capsule with synovium) and blood serum using next generation sequencing, in ageing and OA from young and adult mice, and old mice using a traumatic *in vivo* OA model.

## 2 Methods

All reagents were from ThermoFisher-Scientific unless stated otherwise.

### 2.1 Animals

All animal protocols were performed in accordance with the guidelines (Animals (Scientific Procedures) Act 1986) following ethical review and approval. Four groups of C57BL6/J male mice were used for small RNA sequencing. Old mice were 18 months old (n ​= ​6), young mice 8 months old (n ​= ​6) and mice used for destabilization of the medial meniscus (DMM) 24 months old (Sham n ​= ​3; DMM n ​= ​6) [14]. Mice were group housed in individually ventilated cages at a 12-h light/dark cycle, with *ad libitum* access to food and water.

### 2.2 Surgical induction of OA by DMM in mice

DMM surgery was performed as previously reported [15]. Briefly, anaesthesia was induced with an intramuscular injection of 10 ​μl/g of Hypnorm®/Hypnovel® and mice were maintained under a plane of general anaesthesia using isoflurane during the surgical procedure. A small incision was made over the medial aspect of the patellar tendon, and the joint capsule incised. Using blunt dissection small amounts of fat were removed allowing for visualisation of the medial meniscotibial ligament (MMTL). Using a scalpel, the MMTL was transected using an upwards motion from the cranial horn of the MMTL on the proximal tibial plateau. Once transected, the joint capsule and the skin were sutured. In sham operated mice the MMTL was visualised but not transected. Mice were immediately transferred to a heated post-operative recovery room. All animals received 0.1 ​mg/kg buprenorphine HCl (Vetergesic; Alstoe Animal Health, York, UK) subcutaneously post-surgery. They were monitored daily to ensure they were in good health. The animals were allowed to freely move with unrestricted access to food and water. Mice were sacrificed eight weeks post-surgery by terminal anaesthesia using pentobarbitone.

### 2.3 Joint and serum collection for small RNA sequencing

As OA is a whole joint disease, we undertook our analysis on whole mouse joints including cartilage, meniscus, subchondral bone and joint capsule with synovium. Following euthanasia, knee joints were collected from all young, old, DMM and Sham groups for small RNA sequencing [13]. Briefly, the joints were freed from soft tissues, harvested by cut at 7 ​mm cranially and caudally from the centre of the joint and stored in RNAlater. Blood was collected using cardiac puncture following terminal anaesthesia into plain tubes onto ice and left to clot for 1 ​h. Blood was then spun for 5 ​min at 14000 ​rpm and the serum removed. One serum sample from the old mice group was excluded from further processing due to extensive haemolysis.

### 2.4 Osteoarthritis research society international (OARSI) scoring of histological sections of mouse knee joints

To evaluate the extent of OA, total knee joints were collected from an additional cohort of equivalent aged young (n ​= ​8), old (n ​= ​4), Sham (n ​= ​5) and DMM (n ​= ​6) mice [13]. For these samples, the procedure, surgeon and duration of the studies were identical. In brief, knee joints were collected into 4% paraformaldehyde and decalcified in 0.5 ​M ethylenediaminetetraacetic acid (pH 7.4) for four weeks at 4 ​°C and coronally embedded in paraffin. Samples were sectioned and stained with Safranin-O Fast-Green; histological scoring (defined as the severity and extent of OA) was undertaken by two blinded independent observers using the OARSI scoring system [16]. Using a scale from 1 to 6, all four quadrants of the section (medial tibial plateau, lateral tibial plateau, medial femoral condyle and lateral femoral condyle) were scored individually and added for each histological section. For statistical analyses, the mean summed score values of three to five sections per knee at four depths throughout the joint were determined. Inter-observer variability was calculated using Cohen's Kappa statistics [17].

### 2.5 RNA isolation, cDNA library preparation and small RNA sequencing

Approximately the same amount of joint tissue per donor was pulverised into a powder with a dismembranator (Mikro-S, Sartorius, Melsungen, Germany) under liquid nitrogen, and total RNA was extracted using a miRNeasy kit (Qiagen, Crawley, UK).

Total RNA was extracted from 500 ​μl serum using a RNeasy Serum kit (Qiagen, Crawley, UK) with DNase treatment (Qiagen, Crawley, UK). RNA integrity (RIN) was confirmed using the Agilent 2100 Bioanalyzer (Agilent Technologies, Santa Clara, USA). Ribosomal RNA (rRNA) was depleted using the Ribo-Zero™ rRNA Removal Kit (Epicentre, Madison, USA). Quality control prior to sequencing was performed by Qubit and Bioanalyzer (Agilient Technologies, Santa Clara, USA) analysis with RNA Pico chips and small RNA chips to measure RNA quantity, RNA integrity and calculate microRNA percentage. 100 ​ng of rRNA-depleted RNA per sample were submitted for library preparation using a NEB small RNA library kit (New England Biolabs (NEB), Ipswich, USA) with the addition of tobacco acid pyrophosphatase (Epicentre, Madison, USA). Pooled samples were size selected (120-300bp) and purified with Ampure beads (Agencourt, Beckman-Coulter, High-Wycombe, UK). Sequencing was undertaken on an Illumina HiSeq 2000 platform (Illumina, San Diego, USA) using 100 base paired-end reads.

### 2.6 Small RNA sequencing data analysis

Statistical analysis for the miRNA sequencing data was performed by the Centre for Genomic Research of the University of Liverpool.

Small RNA sequencing data were processed to obtain microRNA expression values. The processes included basecalling and de-multiplexing of indexed reads using CASAVA version 1.8.2; adapter and quality trimming using Cutadapt version 1.2.1 [18] and Sickle version 1.200 to obtain fastq files of trimmed reads. Aligning reads to Ensembl GRCm38.77 mouse genome reference sequences containing 2045 annotated microRNA features using Tophat version 2.1.0 [19] with option “-g 1”; counting aligned reads against transcript features using THSeq-count. Count values for microRNA features were used as microRNA expression measurements for differential expression (DE) analysis.

DE analysis was performed in R environment using edgeR [20] and included: assessing data variation using principal component analysis (PCA) and correlation analysis; handling library size variation respectively for joint samples and serum samples through data normalisation; formulating data variation using negative binomial distributions; modelling data using a generalised linear model; computing log2 Fold Change (logFC) values for required contrasts based on model fitting results through contrast fitting approach, assigning p-values to logFC values by likelihood-ratio [21] testing; dealing with the effects of multiple tests using Benjamini-Hochberg [22] approach to obtain false discovery rate (FDR) values; and defining significantly DE microRNAs as those with FDR ​< ​5%.

Sequence data available at National Centre for Biotechnology Information Gene Expression Omnibus; *E*-MTAB-4878.

### 2.7 RNA isolation, poly(A) cDNA synthesis and microRNA qRT-PCR

Validation of the selected microRNA sequencing results of mice knee joints was undertaken using qRT-PCR in the discovery cohort used for sequencing and an independent (validation) cohort of mice; the latter consisting of six adult (7 months) and six old (24 months) mice.

Total RNA for the discovery cohort was extracted as described above for isolation of RNA for small RNA sequencing. For the validation cohort, RNA was extracted using TRIzol reagent. Poly(A) cDNA synthesis was completed using 200 ​ng RNA and miScript II RT Kit and diluted in 180 ​μl RNAse-free water. qPCR was completed using miScript SYBR Green PCR kit. MicroRNA expression data was normalised to a validated housekeeping gene. DE microRNAs were selected for further validation based on level of differential expression and following a literature review. These were miR-140–5p, miR-205–5p, miR-682, miR-499–5p, miR-455–3p, miR-6238, miR-455, miR-146a-5p and miR-151–5p. Qiagen miScript Primer Assays used can be found in Supplementary file 1.

### 2.8 MicroRNA target prediction and pathway analysis

Bioinformatic analysis was performed using Ingenuity Pathway Analysis (IPA) software (IPA, Qiagen Redwood City, CA, USA), to identify relationships, mechanisms, functions and pathways associated with DE microRNAs, as well as identifying putative messenger RNA (mRNA) targets.

DE microRNA data was uploaded into the ‘MicroRNA Target Filter’ module in IPA. A conservative filter was applied (only experimentally validated and highly conserved mRNA targets in mice). These were further filtered on available cell types most representative of the whole joint; chondrocyte, osteoblasts, fibroblasts, bone marrow cells and skeletal muscle.

Multiple ‘Core Analysis’ were performed using DE microRNAs and their predicted mRNA targets, between young vs. old; Sham vs. DMM; and old vs. DMM joint tissues, querying for associated diseases, molecular and cellular functions, canonical pathways, novel networks and common upstream regulators.

### 2.9 Statistical analysis

Histological scoring was tested for normality and evaluated via a non-parametric Mann-Whitney-U test, using GraphPad Prism version 8.0 (GraphPad Software, La Jolla California USA, www.graphpad.com). Inter-observer agreement of histological scores was calculated using Cohen's kappa coefficient. The sample correlation heatmap was constructed using the R package edgeR [20]. PCA plots were created using Metaboanalyst [23]. Relative gene expression was calculated using the 2^-DCT method. For statistical evaluation of gene expression data, and following normality testing, two-tailed t-tests or Mann-Whitney U tests were performed in GraphPad Prism version 8.0 for Windows (GraphPad Software, La Jolla California USA, www.graphpad.com); a cut-off for statistical relevance was made at p-value 0.05.

## 3 Results

### 3.1 OARSI scoring of joints

**Fig. 1:**
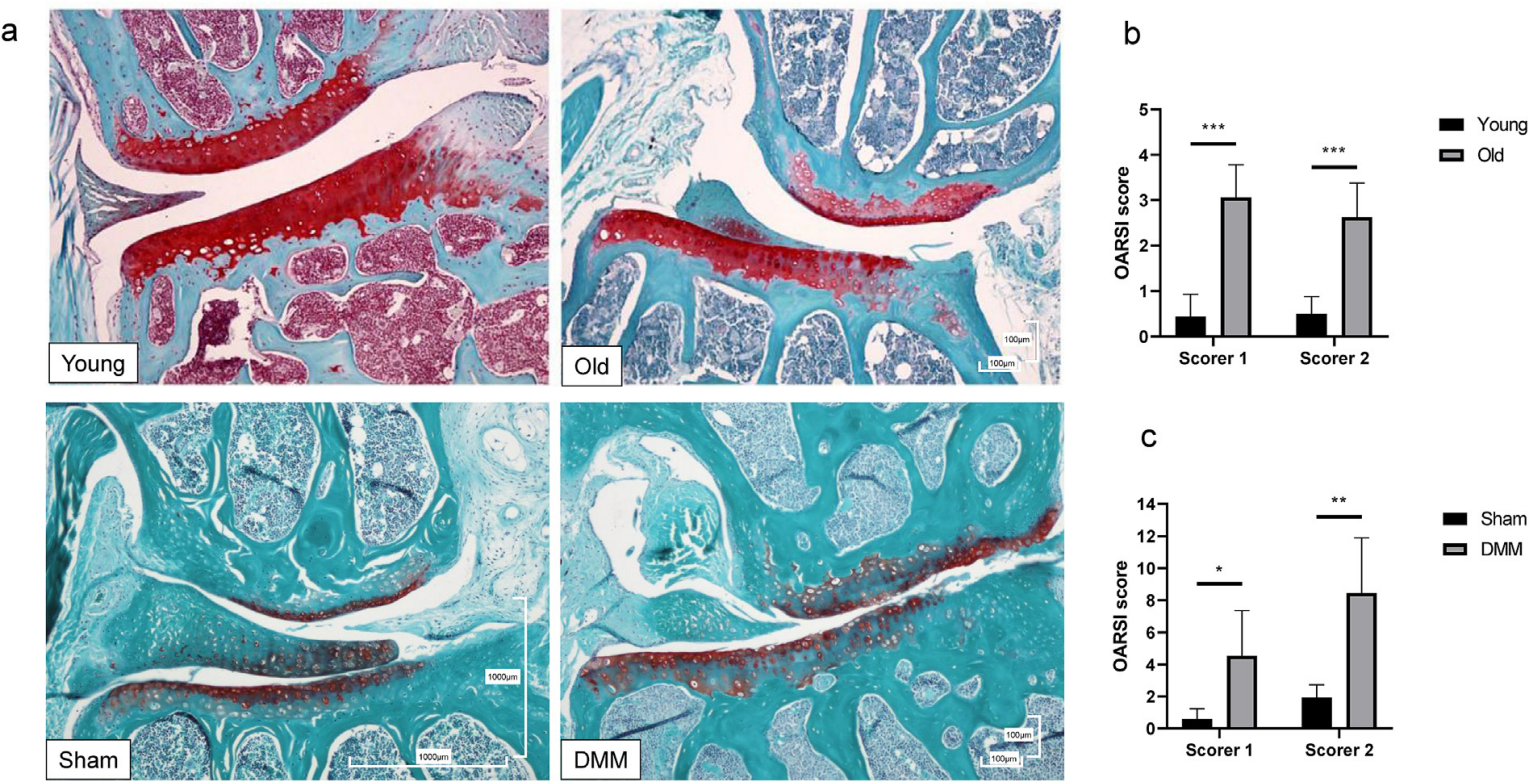
Histological changes in the mouse knee and assessment of osteoarthritis using OARSI scores, as previously shown in Steinbusch et al., 2017 [13]. (a) Histological images showing the medial femoral condyle (above) and medial tibial plateau (below) for young, old, Sham and DMM mice knees. Safranin-O with Fast-Green counterstain; red indicates proteoglycan. Scale bar 100 ​μm. (b/c) Assessment of osteoarthritis using OARSI scores in young (n ​= ​8) vs. old (n ​= ​4) (b); and in Sham (n ​= ​5) vs. DMM (n ​= ​6) (c). Data represents the mean ​+ ​95% confidence interval (CI) for each scorer. ∗p ​< ​0.05, ∗∗p ​< ​0.01, ∗∗∗p ​< ​0.001. (For interpretation of the references to colour in this figure legend, the reader is referred to the Web version of this article.)

Histological images and OARSI scoring of joints for this group of mice have been previously described [13]. In brief, mice exhibited typical histological features of OA in the DMM knees such fibrillations and loss of staining. OARSI scoring (mean ​± ​95% confidence interval (CI)) for young and old were 0.5 ​± ​0.3 and 2.8 ​± ​2.7 (p ​= ​0.01), and Sham and DMM were 1.25 ​± ​1.1 and 6.5 ​± ​0.7 (p ​< ​0.001), respectively. Cohen Kappa's statistic was 0.4 indicating a fair agreement between observers. DMM mice exhibited typical OA histological features (Fig. 1a).

#### 3.1.1 Preliminary analysis of small RNA sequencing data

**Fig. 2:**
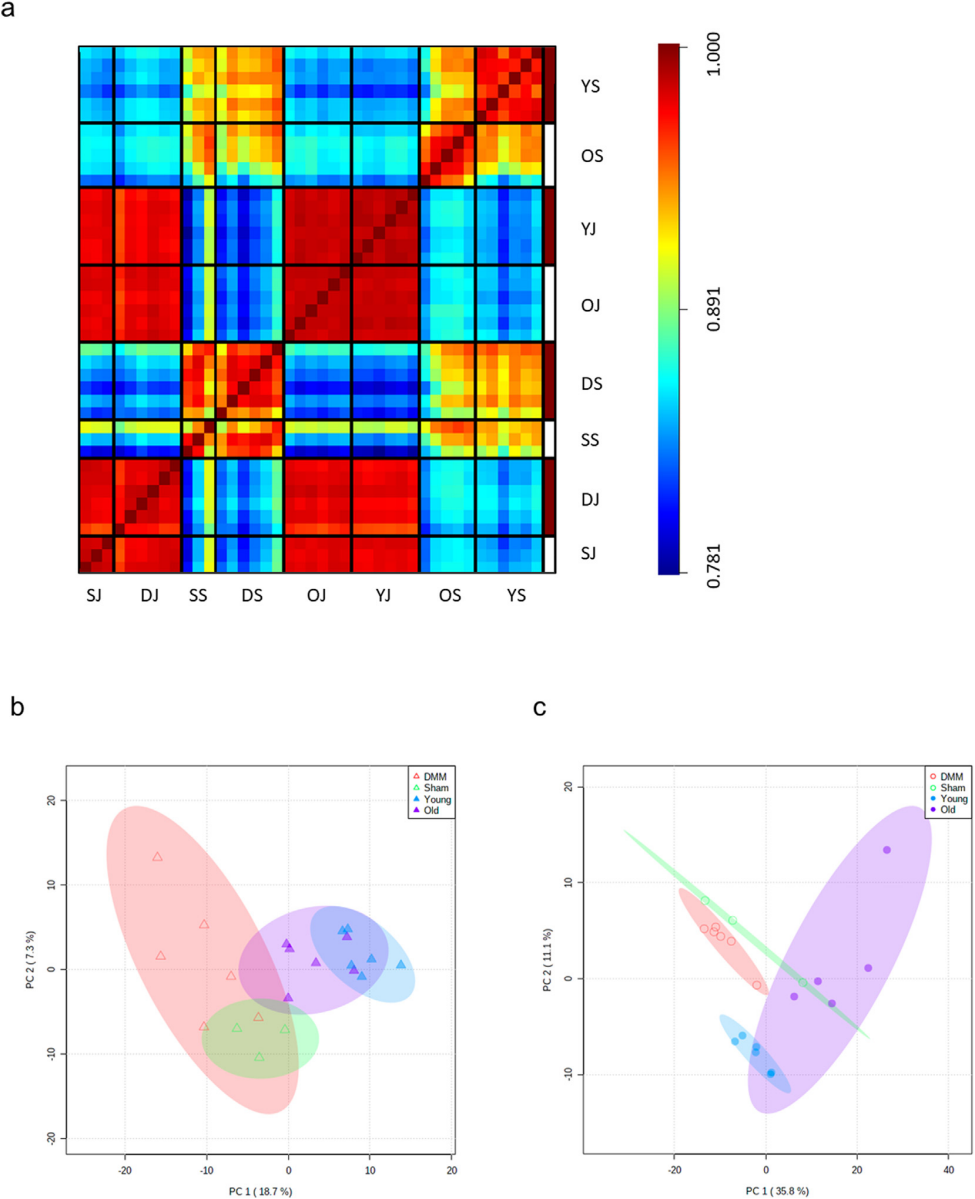
(a) Sample correlation heatmap illustrating the relationship between each microRNA dataset compared to every other dataset. Colours represent the degree of correlation between comparisons, ranging from blue (weakest correlation) to red (strongest correlation). Abbreviations; young joint (YJ), old joint (OJ), Sham joint (SJ), destabilization of the medial meniscus/DMM joint (DJ), young serum (YS), old serum (OS), Sham serum (SS) and destabilization of the medial meniscus/DMM serum (DS). (b, c) Principal component analysis (PCA) plots of logarithm-transformed microRNA abundance data for PC1 vs. PC2 in (b) joint and (c) serum samples. Coloured regions demark 90% confidence regions. Colour difference separates groups (DMM, Sham, young and old); marker fullness separates cohorts (cohort 1, DMM/Sham; cohort 2, young/old). Legends to the main features are shown. Abbreviations; destabilization of medial meniscus (DMM). (For interpretation of the references to colour in this figure legend, the reader is referred to the Web version of this article.)

To identify DE microRNAs in mouse joints and serum in response to age and OA, 41 cDNA libraries representing old and young joints and serum (old joint ​= ​OJ; young joint ​= ​YJ; old serum ​= ​OS; young serum ​= ​YS), and Sham and DMM joints and serum (Sham joint ​= ​SJ; DMM joint ​= ​DJ; Sham serum ​= ​SS; DMM serum ​= ​DS) were constructed. Prior to library preparation, RNA integrity of joint tissues was assessed and revealed RIN values of (average; std dev) 7.1 ​± ​0.8. Libraries were subjected to Illumina sequencing and after setting a baseline of 10 counts per million reads to filter out noise, a total of 763 microRNAs across all libraries were identified. These results were used in a sample correlation heatmap (Fig. 2a). Joint samples showed a strong correlation with other joint samples and a weaker correlation with serum samples; serum samples showed some degree of correlation with other serum samples, although this was not as strong as the correlation between joint samples. This shows that microRNA datasets vary much more between serum samples than between joint samples, and that the correlation between serum and joint samples is not strong.

#### 3.1.2 Identification of DE microRNAs using small RNA sequencing

In joint tissues (Table 1), miR-140–5p, miR-205–5p, miR-682, miR-208b-3p, miR-499–5p, miR-455–3p and miR-6238 were DE between the young and old groups; miR-146a-5p, miR-3474, miR-615–3p and miR-151–5p were DE between DMM and Sham groups; and miR-652–3p, miR-23b-3p, miR-708–5p, miR-5099, miR-23a-3p, mir-214–3p, miR-6238 and miR-148–3p were DE between the old and DMM groups.

**Table 1:** Differentially expressed microRNAs in joint tissues between contrasts and their log2 fold changes.

Contrast	DE microRNAs	Accession number	logFC	FDR-adjusted p value	Meaning
Young vs. Old	miR-140–5p	MIMAT0000151	0.939	0.0379	Higher in young
	miR-205–5p	MIMAT0000238	−3.658	0.0379	Lower in young
	miR-682	MIMAT0003459	−1.430	0.0379	Lower in young
	miR-208b-3p	MIMAT0004939	2.55	0.0261	Higher in young
	miR-499–5p	MIMAT0003482	1.772	0.0025	Higher in young
	miR-455–3p	MIMAT0003742	1.352	0.00010	Higher in Young
	miR-6238	MIMAT0024859	−2.198	0.00007	Lower in Young
DMM vs. Sham	miR-146a-5p	MIMAT0000158	2.706	0.0362	Higher in DMM
	miR-3474	MIMAT0015646	−3.124	0.0362	Lower in DMM
	miR-615–3p	MIMAT0003783	−1.523	0.0048	Lower in DMM
	miR-151–5p	MIMAT0004536	3.241	0.0048	Higher in DMM
Old vs. DMM	miR-652–3p	MIMAT0003711	−0.903	0.0461	Lower in old
	miR-23b-3p	MIMAT0000125	−0.920	0.0231	Lower in old
	miR-708–5p	MIMAT0004828	−1.058	0.0268	Lower in old
	miR-5099	MIMAT0020606	−1.081	0.0044	Lower in old
	miR-23a-3p	MIMAT0000532	−1.083	0.0063	Lower in old
	miR-214–3p	MIMAT0000661	−1.157	0.0192	Lower in old
	miR-6238	MIMAT0024859	−1.433	0.0192	Lower in old
	miR-184–3p	MIMAT0000213	−3.479	0.0089	Lower in old

Abbreviations; Differentially expressed (DE), log2 fold change (logFC), false discovery rate (FDR).

The number of DE microRNAs in serum was much higher, and further details can be found in Supplementary file 2. In brief, we identified 214 DE microRNAs in serum between young and old mice, two of which were also DE in joint tissues – miR-140–5p and miR-455–3p; 13 DE microRNAs were identified between Sham and DMM groups, yet none of these were DE in the respective joint samples; and of the 197 DE microRNAs between old and DMM in serum, three were also DE in joint samples – miR-23b-3p, miR-5099 and miR-6238.

The effect of group classification on the expression of microRNAs in joint tissues can be observed by the partial separation of clusters on the 1st component. Whilst the separation between DMM and Sham was weak, both clearly separated from young and old samples (Fig. 2b). Samples from DMM joint tissues were the most variable. For serum samples, the PCA plot indicated better separation of clusters (Fig. 2c).

### 3.2 Validation of small RNA sequencing data using RT-qPCR

Nine microRNAs were selected for further validation based on level of differential expression (FDR<0.05, logCPM>3) and previous literature; these included microRNAs that were upregulated in young mice (miR-140–5p, miR-499–5p, miR-455–3p and miR-455), microRNAs that were downregulated in young mice (miR-205–5p, miR-682 and miR-455) and some microRNAs that were not differentially expressed in young vs old but were DE in Sham vs DMM and deemed relevant based on previous literature (miR-151–5p and miR-146a-5p) [24,25].

**Fig. 3:**
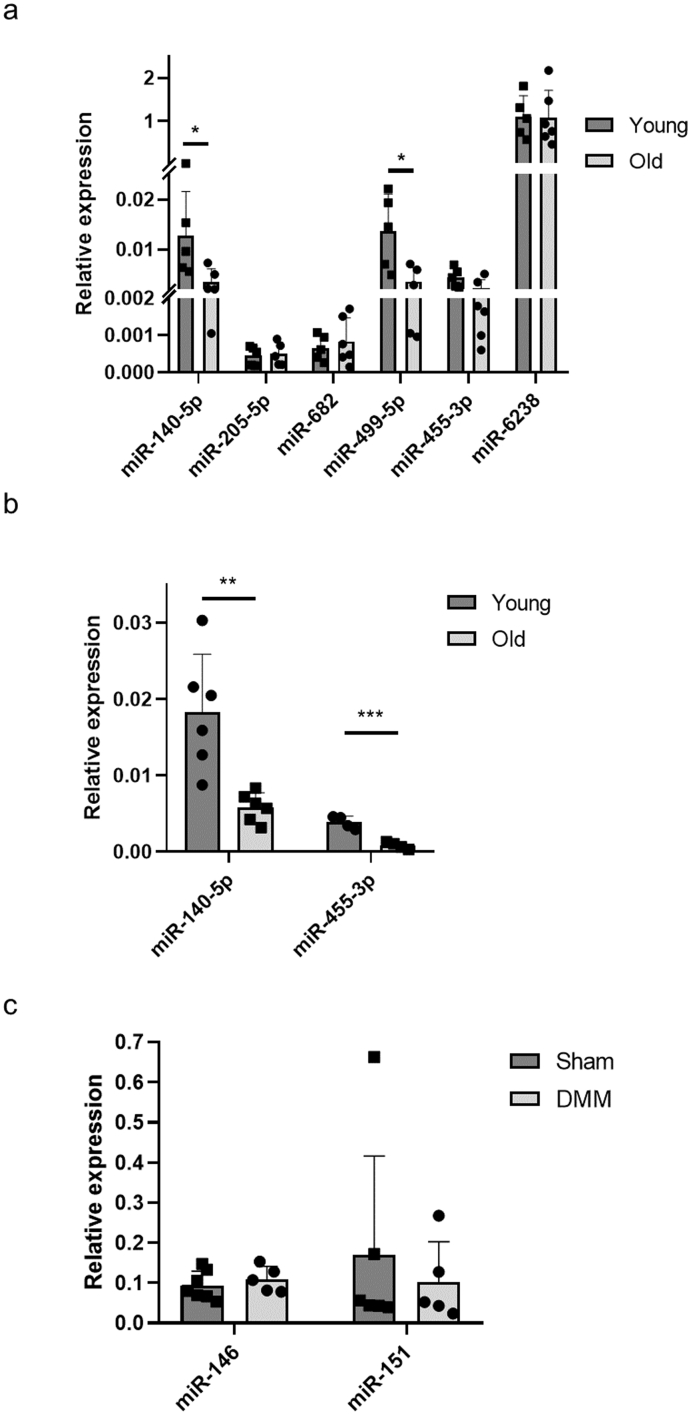
Validation of differentially expressed microRNAs between young vs. old samples following small RNA sequencing in discovery (a) and validation (b) cohorts using RT-qPCR. Histograms of relative expression calculated using 2^-DCT method using U6 as an endogenous control. Bars represent means with standard deviation. ∗p ​< ​0.05, ∗∗p ​< ​0.01, ∗∗∗p ​< ​0.001. (c) RT-qPCR results for two of the differentially expressed microRNAs between Sham vs. DMM samples following small RNA sequencing in the discovery cohort. Histograms of relative expression calculated using 2^-DCT method using U6 as an endogenous control. Bars represent means with standard deviation.

Validation was carried through RT-qPCR using the discovery cohort and a validation cohort (comprised of two groups: young n ​= ​6, 7 months-old; and six old n ​= ​6, 24 months-old) of joint samples. In agreement with sequencing data, miR-140–5p and miR-499–5p were significantly upregulated in the young group when compared to the old group in the discovery cohort (Fig. 3a). MiR-140–5p was also significantly increased in young samples compared to old samples in the validation cohort, alongside miR-455–3p (Fig. 3b). Although not statistically significant, miR-205–5p and miR-682 were downregulated in young samples in accordance with sequencing results (Fig. 3a).

When comparing Sham vs. DMM in the discovery cohort, RT-qPCR failed to validate two microRNAs; miR-146a-5p and miR-151–5p (Fig. 3c).

#### 3.2.1 MicroRNA target predictions and pathway analysis

IPA predicted 2210 ​mRNA targets for the DE microRNAs (identified by sequencing) across all comparisons. (Supplementary file 3).

**Fig. 4:**
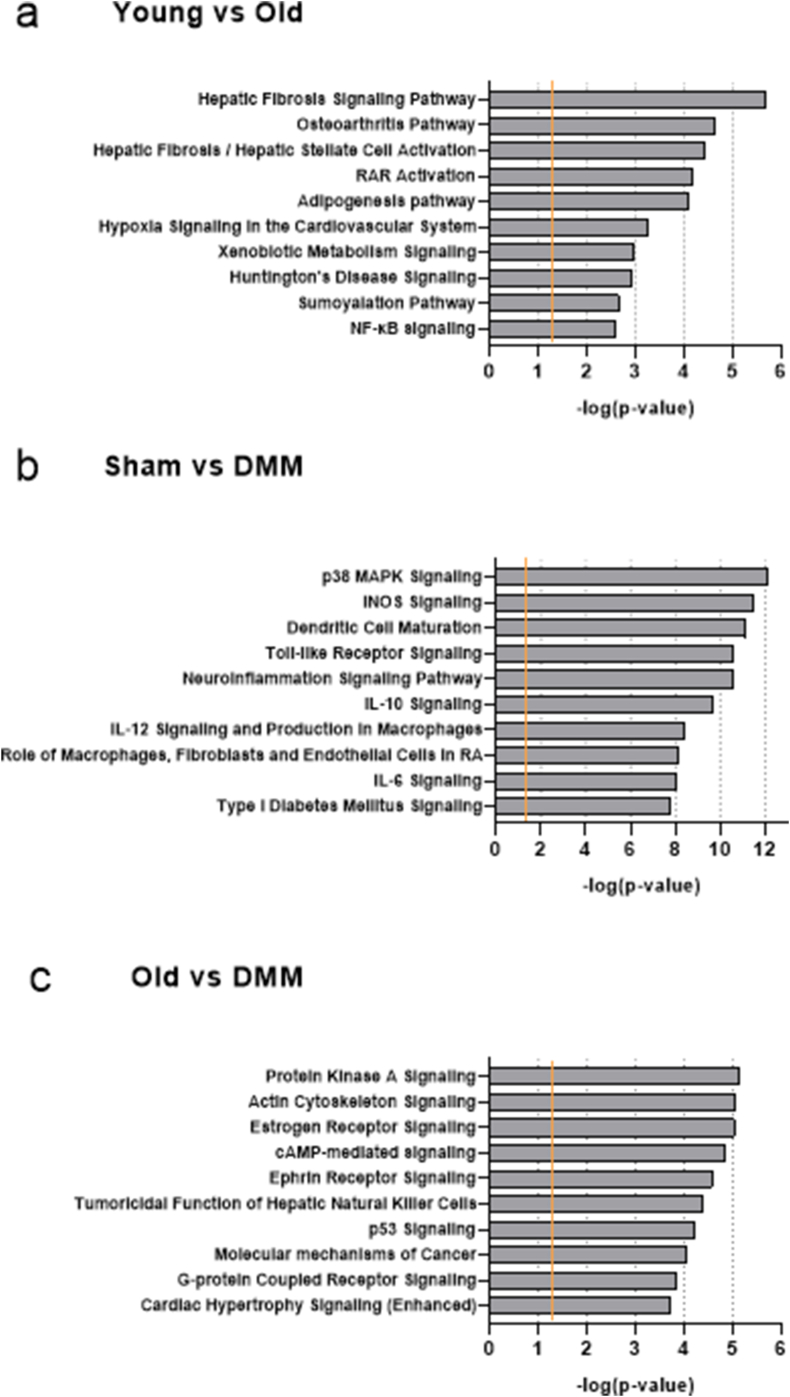
Top 10 Ingenuity Pathway Analysis (IPA) predicted canonical pathways from differentially expressed microRNAs and their respective predicted mRNA targets in joint tissues for young vs. old, Sham vs. DMM and old vs. DMM joint tissues. Orange line represents the cut-off for statistical relevance at p ​< ​0.05, corresponding to -log (p-value) ​≈ ​1.3. Abbreviations; destabilization of the medial meniscus (DMM). (For interpretation of the references to colour in this figure legend, the reader is referred to the Web version of this article.)

Multiple ‘Core Analysis’ were performed using DE microRNAs and their predicted mRNA targets between young vs. old; Sham vs. DMM; and old vs. DMM joint tissues. Fig. 4 shows the top 10 predicted canonical pathways associated with DE microRNAs and predicted mRNA targets for all datasets.

When comparing young and old joint tissue samples, we found 12 molecules associated with the osteoarthritis pathway (p ​= ​2.4 ​× ​10^−5^) (Fig. 4a). Nuclear factor kappa B (NF-kB) signalling presented as the 10th most likely pathway. IPA also identified associated cellular functions related to bone and cartilage morphology and function, including bone mineral density (p ​= ​2.38 ​× ​10^−6^), cartilage development (p ​= ​1.07 ​× ​10^−3^), differentiation of chondrocytes (p ​= ​4.75 ​× ​10^−8^), differentiation of osteoblasts (p ​= ​5.37 ​× ​10^−6^), limb development (p ​= ​3.22 ​× ​10^−4^) and also inflammatory response (p ​= ​1.11 ​× ​10-3); Supplementary figure 4. Shows the networks pertaining to these functions, including microRNAs and predicted mRNAs involved.

Analysis of DE molecules between Sham and DMM joint tissues revealed different canonical pathways relevant to osteoarthritis, including p38 MAPK signalling (p ​= ​7.85 ​× ​10^−13^); inducible nitric oxide synthase (iNOS) signalling (p ​= ​3.18 ​× ​10^−12^); and interleukin (IL) signalling pathways such as IL-10 (p ​= ​2.3 ​× ​10^−10^) and IL-6 (p ​= ​8.23 ​× ​10^−9^) signalling. Other interesting canonical pathways not represented in Fig. 4a included NF-kB signalling (p ​= ​2.9 ​× ​10^−8^) and IL-1 signalling (p ​= ​9.74 ​× ​10^−7^). Significant cellular functions related to cell-mediated immune response, inflammation and bone structure were predicted by IPA, including inflammatory response (p ​= ​7.23 ​× ​10^−11^), adhesion of immune cells (p ​= ​4.09 ​× ​10^−11^), fibrosis (p ​= ​1.24 ​× ​10^−20^), quantity of bone (p ​= ​2.05 ​× ​10^−13^) and resorption of bone (p ​= ​6.75 ​× ​10^−8^); (Supplementary figure 5).

In relation to old and DMM joint tissues, the top 10 inferred canonical pathways included Protein Kinase A signalling (p ​= ​7.39 ​× ​10^−6^), oestrogen receptor signalling (p ​= ​9.26 ​× ​10^−6^) and p53 signalling (p ​= ​6.12 ​× ​10^−5^). Significant cellular functions inferred were related to musculoskeletal development and abnormalities, including abnormal morphology of skeletal muscle (p ​= ​1.53 ​× ​10^−3^), function of muscle (p ​= ​4.76 ​× ​10^−5^), abnormal morphology of bone (p ​= ​3.07 ​× ​10^−5^), differentiation of osteoblasts (p ​= ​4.96 ​× ​10^−4^) and activation of bone cells (p ​= ​2.45 ​× ​10^−4^) (Supplementary figure 6).

## 4 Discussion

MicroRNA expression profiling is useful in identifying regulatory molecules with relevant functions in many diseases, including OA [26]. While ageing is the main risk factor for the development of OA, few studies interrogate microRNA expression in ageing tissues. Our study investigated the microRNA profile of mice whole joints and serum in ageing and OA, as well as their functional significance for the development and progression of disease using a mouse model of post-traumatic OA. Results revealed a pattern of microRNA DE between Sham and DMM groups as well as young and old mice: miR-140–5p, miR-205–5p, miR-682, miR-208b-3p, miR-499–5p, miR-455–3p and miR-6238 were DE between the young and old groups; miR-146a-5p, miR-3474, miR-615–3p and miR-151–5p were DE between DMM and Sham groups; and miR-652–3p, miR-23b-3p, miR-708–5p, miR-5099, miR-23a-3p, Mir-214–3p, miR-6238 and miR-148–3p were DE between the old and DMM groups. The precise mechanisms behind these alterations are still unknown, yet predicted cellular functions associated with the DE microRNAs appear to be related to bone and cartilage morphology and function, cell-mediated immune response, inflammation and musculoskeletal development and abnormalities. Because microRNAs are highly conserved between species it is likely that their expression is altered in humans in a similar fashion; further research into these specific molecules in human tissues is now warranted to check their potential suitability as biomarkers of ageing and disease.

The DMM mouse model is a post-injury model of good reproducibility that closely resembles human OA [15]. To investigate the effects of age we chose mice according to their human equivalent age – young 8-month-old mice, which are skeletally mature and comparable to 25-28-year-old humans; and 18-24-month-old mice which are comparable to 40-50-year-old humans, as OA most commonly affects people over the age of 40 [27]. Histological analysis revealed a mild OA-like pathology in the old and Sham mice, indicating early stages of naturally occurring OA similar to that in human knees of equivalent age [28]. DMM surgery exacerbated the severity of OA when compared to Sham, indicating the procedure was successful in recreating an OA-like environment.

While pathological processes can target a single joint tissue, ultimately all joint tissues are affected due to their functional association and physical proximity, thus rendering OA a whole-joint disease [29]. As such, small RNA sequencing was undertaken on the entire mouse joints as opposed to single tissues. Approaching the whole organ might be less sensitive in detecting gene alterations in single tissues and might limit the ability to determine which particular tissue contributed to expression of a specific gene, however it has the advantage of allowing discovery of genes more globally involved in OA progression. A total of 763 microRNAs across all libraries were identified; similar numbers of microRNAs have been identified in previous studies [30,31]. Differences in joint microRNA profiles could be correlated to the animals’ disease state and/or age, with a total of 19 microRNAs DE between young and old; Sham and DMM; and old and DMM.

Among the DE microRNAs found in our study, miR-140–5p was significantly increased in healthy young compared to old. MiR-140 has been extensively investigated as a regulator of cartilage homeostasis, particularly chondrocyte proliferation [32]. It targets multiple genes that inhibit cartilage degradation, such as matrix metalloproteinase 13 (MMP-13) [33] and A Disintegrin-like and Metalloproteinase with Thrombospondin Type 1 Motif 5 (ADAMTS5) [33]; and its expression is reduced in OA cartilage [34]. As miR-140–5p was downregulated in old healthy mice, our study suggests that this microRNA is not only involved in the OA pathway, but also in physiological ageing. It is possible that changes previously attributed to OA could in fact be due to age. For example, in the aforementioned study by Miyaki et al. [34], miR-140–5p expression was compared between eight normal donors, mean age ​± ​SD of 38.33 ​± ​5.31 years and 11 OA patients, mean age ​± ​SD of 79.36 ​± ​9.72 years, Differences in expression were attributed to the presence of OA, however the age differences between groups must be taken into consideration given its critical role in OA development. The effect of age on miR-140–5p expression cannot be excluded unless comparisons are made between age-matched groups. To the best of our knowledge, ours is the first study to show a direct association between a decrease in miR-140–5p expression and healthy ageing joint tissues, supporting the premise that ageing shares regulatory networks with OA. Our findings also suggest that miR-140–5p might be involved in age-related, naturally occurring OA. This is in line with the results from a previous study that reported that miR-140 deletion in healthy mice leads to the development of age-related OA-like pathology that is not observed in age-matched wild-type mice [35]. Moreover, the host-miRNA relationship between Wwp2 and miR-140 is evolutionarily conserved among vertebrates and the possibility remains that these two factors cooperate not only in the context of cartilage homeostasis but also pathogenic processes such as osteoarthritis [36]. Interestingly, our sequencing results showed serum miR-140–5p levels to actually be increased in older animals, as opposed to what appears to be happening in the joint; while these results require further validation, we hypothesise that unknown mechanisms are actively increasing transportation of these particular miRNAs out of the joint environment and into the bloodstream.

MiR-499–5p expression was increased in young mice compared to the old. This microRNA, currently referred to as miR-499a, regulates chondrogenesis in human bone marrow derived mesenchymal stem cells [37]. MiR-499a is increased in OA human cartilage, particularly in late-stage OA, suggesting miR-499a levels may be associated with OA progression [38]. Upregulation of miR-499a promotes chondrocyte extracellular matrix degradation by supressing growth differentiation factor-5 (GDF5) [38]. Inhibition of mir-499a in an *in vivo* OA rat model promoted cartilage regeneration and prevented progression [39].

Expression of miR-455–3p was increased in young mice compared to old. MiR-455–3p regulates early chondrogenic differentiation in ATDC5 cells by inhibiting runt-related transcription factor 2 (RUNX2) expression [40]. It also regulates DNA methylation during chondrogenic differentiation of bone marrow derived stem cells, attenuating cartilage degeneration [41]. In human chondrocytes miR-455–3p promotes TGF-β signalling and inhibits OA development by targeting P21-activated kinase 2 (PAK2) [42]; however in this study there was a ~44 year age difference between healthy and OA cohorts and therefore we cannot ascertain whether age or OA impacts miR-455–3p expression. Our results suggest that similarly to miR-140–5p, miR-455–3p expression is related to both physiological ageing and disease state, potentially impacting networks that lead to age-related OA. miR-455–3p expression was also significantly increased in serum of younger animals; while further validation is required, comparing serum levels of miR-455–3p between healthy ageing and disease could provide new insights into the pathophysiology of OA.

Pathway analysis of DE microRNAs in joint tissues revealed OA-related pathways in all group comparisons (young vs. old, Sham vs. DMM, old vs. DMM), including NF-kB, p38 MAPK and iNOS. Interestingly, many of these pathways have also been linked to ageing, highlighting the commonality of networks driving ageing and OA. For example, NF-kB which is one of the best-characterised signalling pathways activated by OA stimuli [43] also appears a key mediator of the low-level inflammatory state known as inflamm-ageing [44].

p38 MAPK signalling has been reported to play a significant role in the progression of OA as its activation promotes overexpression of proinflammatory cytokines, chemokines and signalling enzymes in human OA chondrocytes [45], yet this pathway is also involved musculoskeletal processes of ageing such as autonomous loss of satellite cell self-renewal in aged skeletal muscle in mice [46]; and while OA joints display increased levels of markers of nitric oxide (NO) production [47], there is growing evidence that chondrocyte NO levels increase with age and are accompanied by a decrease in anti-oxidant capacity, thus altering cartilage homeostasis and contributing to OA development [48].

OA is a multidimensional disease regulated by an extremely complex network. Identifying transcription factors that control gene expression in ageing and OA is vital to determine potential OA biomarkers and develop novel therapies. Prospective intra-articular microRNA treatments have emerged in recent years; an example is miR-140–5p which, as previously mentioned, has been shown inhibit inflammation and stimulating chondrogenesis *in vitro.* In a study by Si et al., 2017 [33] a miR-140 mimic was administered intra-articularly in OA rats and was shown to alleviate OA progression by modulating extracellular matrix homeostasis, with no complications associated with the route of administration. A more recent study by Tao et al. [49] has also shown that administering exosomes derived from miR-140-5p-overexpressing human synovial mesenchymal stem cells intra-articularly enhances cartilage regeneration and prevents knee OA in a rat model; similarly, Gent et al. [50] showed that intra-articular injection of human umbilical cord mesenchymal stem cells (hUC-MSCs) overexpressing miR-140–5p in a DMM rat model promoted cartilage injury healing. Interestingly, in our study miR-140–5p was significantly decreased in old mice when compared to young; whether this is one of the predisposing factors for old mice to develop OA warrants further investigation. Future research should also investigate whether miR-140 mimic injection could prevent the development of OA in old, healthy mice that already display decreased levels of this molecule. While comprehensive studies are still required to examine the clinical potential of microRNAs for treatment of OA, results thus far are promising and intra-articular administration of microRNAs may offer a safe alternative therapy with few systemic adverse effects. And while miR-140–5p appears to be a strong candidate, other microRNAs have also been investigated; for example, Nakamura et al., 2018 [49] demonstrated that intra-articular injection of locked nucleic acid miR-181a-5p antisense oligonucleotides induced cartilage-protective effects in a rat model of facet joint OA, thus attenuating cartilage degradation. This highlights the importance of validating potential targets of any DE microRNAs to help ascertain their relationship with the development of OA and potentially provide new targets for treatment.

There were some limitations to this study. While DMM is a standard method for establishing an animal model for OA, it naturally differs from the processes by which human OA develops and further analysis on human tissues is desirable. One limitation was that the design was not linear and old healthy mice were not the same age as the “old” Sham and DMM groups. Additionally, we did not undertake the DMM model in young mice or performed this model with female mice, which would have provided us with supplementary information on the role of ageing and the influence of sex on it [6]. Due to the limited availability of samples we were only able to include one timepoint at 8 weeks post-surgery; inclusion of further timepoints post-surgery could have provided further information regarding changes in the very onset of OA. Considering these limitations in sample size, the authors considered it more important to include a bigger number of OA mice, as bigger variability was expected in the microRNA profile in the diseased state rather than the control mice.

Despite some limitations in sample size, according to a study by Baccarella et al. [51] even though using sample numbers below six per group reduces RNA sequencing performance, the number of genes called significant increases as the sample number increases; this means that in the case of pipelines such as the one we used, by having a slightly underpowered approach we are more likely to underestimate rather than overestimate the number of differentially expressed microRNAs. Thet fact that despite this we were able to validate our findings in an independent validation cohort solidifies our findings.

Future work would benefit from validating the predicted mRNA targets in joint tissues and their pathways, allowing for selection of specific molecules that could be targeted for the prevention of OA. Sequencing joint tissues separately might help understand the specific roles of each tissue in the pathophysiology of OA, as well as their individual contributions to the general microRNA profile. Investigating the significantly altered microRNAs between old and DMM groups could provide valuable insight on mechanisms that are unique to OA regardless of age; indeed, our group is currently working on a microRNA analysis between old intact and old damaged human cartilage (data not published).”

Further investigation into circulating serum microRNAs could also be of great relevance; previous studies have identified miR-30c-5p, miR-30b-5p and let-7a-5p to be significantly increased with age [52] which is in agreement with our sequencing results. Validating these results using an independent validation cohort could provide insights into the utility of serum microRNAs as non-invasive biomarkers of OA.

## Author contributions

Mandy J Peffers, Catarina I G D Castanheira, Louise House, Katarzyna Goljanek-Whysall and James Anderson; data analysis and interpretation, manuscript writing, final approval of the manuscript; Peter D Clegg. And Mandy J Peffers; conception and design, manuscript writing, final approval of the manuscript. Yongxiang Fang; assembly of data, data analysis, manuscript writing, final approval of the manuscript. Peter I Milner.; provision of study material, assembly of data, manuscript writing and final approval of the manuscript.

## Role of the funding source

Mandy J Peffers is funded through a 10.13039/100010269Wellcome Trust Intermediate Clinical Fellowship (107471/Z/15/Z). This work was also supported by the 10.13039/501100000265MRC and 10.13039/501100012041Versus Arthritis as part of the 10.13039/501100000265Medical Research Council 10.13039/501100012041Versus Arthritis 10.13039/501100001271Centre for Integrated Research into Musculoskeletal Ageing (CIMA) [MR/R502182/1]. The MRC Versus Arthritis Centre for Integrated Research into Musculoskeletal Ageing is a collaboration between the Universities of Liverpool, Sheffield and Newcastle.

## Declaration of competing interest

The authors declare no competing interests.

## Figures and Tables

**Fig. S1:**
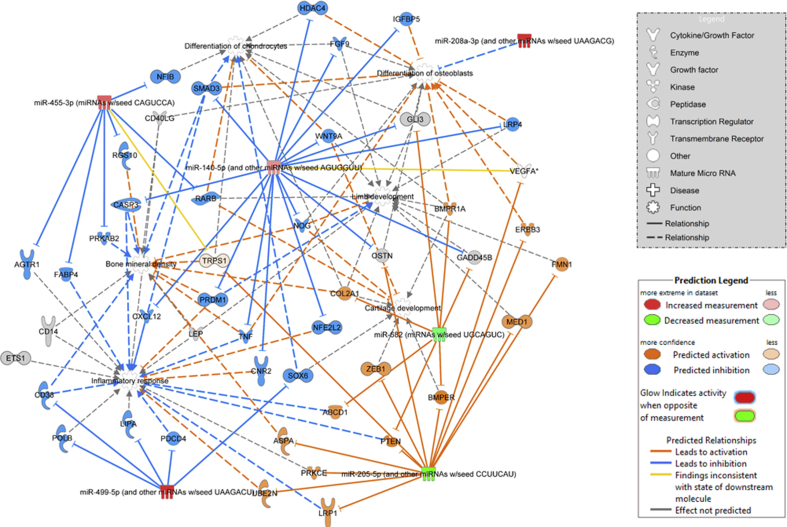


**Fig. S2:**
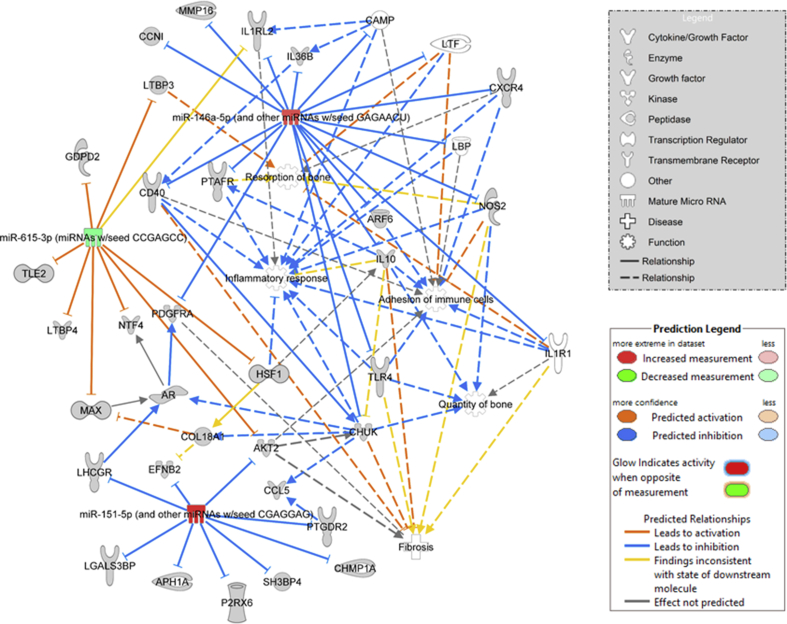


**Fig. S3:**